# Optic neuritis after mRNA COVID‐19 vaccination: a case report

**DOI:** 10.1002/ccr3.8263

**Published:** 2023-11-25

**Authors:** Raghad Tarcha, Afraa Ghazal, Lama Al‐Darwish, Hoda Abdoh, Maysoun Kudsi

**Affiliations:** ^1^ Department of Rheumatology, Faculty of Medicine Damascus University Damascus Syria; ^2^ Al‐Sham Private University (ASPU) Damascus Syria

**Keywords:** case report, COVID‐19 vaccination, optic disc edema, optic neuritis

## Abstract

Our case reported a 22‐year‐old male presented with headache, and sudden vision loss, 10 days after receiving the first dose of COVID‐19 vaccination. Counting fingers in both eyes was his visual acuity on examination and bilateral optic disc edema on fundoscopy was found. Brain MRI was normal. After methylprednisolone pulse therapy, plasmapheresis, and IV cyclophosphamide courses, the optic disc edema disappeared, and his visual function did not improve. Reported cases of optic neuritis develop after mRNA COVID‐19 vaccination are limited.

## Graphical abstract

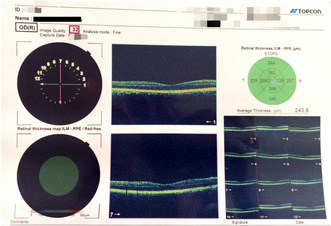

## 1 INTRODUCTION

Although vaccination is an important and effective method to overcome the COVID‐19 disease, flares or new onset of many autoimmune diseases have been described.^1^ Optic neuritis (ON) presented by sudden visual loss and orbital pain. Many factors may play a role in its pathogenesis like trauma, demyelination, infection, autoimmunity, and granulomatous.^2^ Previous researches revealed that some vaccinations against influenza, polio, tetanus, and others may cause ON.^3^

We present a case of bilateral ON, leading to blindness related to the mRNA COVID‐19 vaccination.

## 2 CASE PRESENTATION

A 22‐year‐old male, non‐smoker, non‐alcoholic, was seen due to headache and sudden loss of vision in both eyes and headache. Ten days before these symptoms, he received the first dose of m RNA COVID‐19 vaccination, and at that time, he complained of malaise, low grade fever and arm pain, persisted for 3 days. COVID‐19 polymerase chain reaction test was negative.

He had no previous medical history or family history.

**FIGURE 1:**
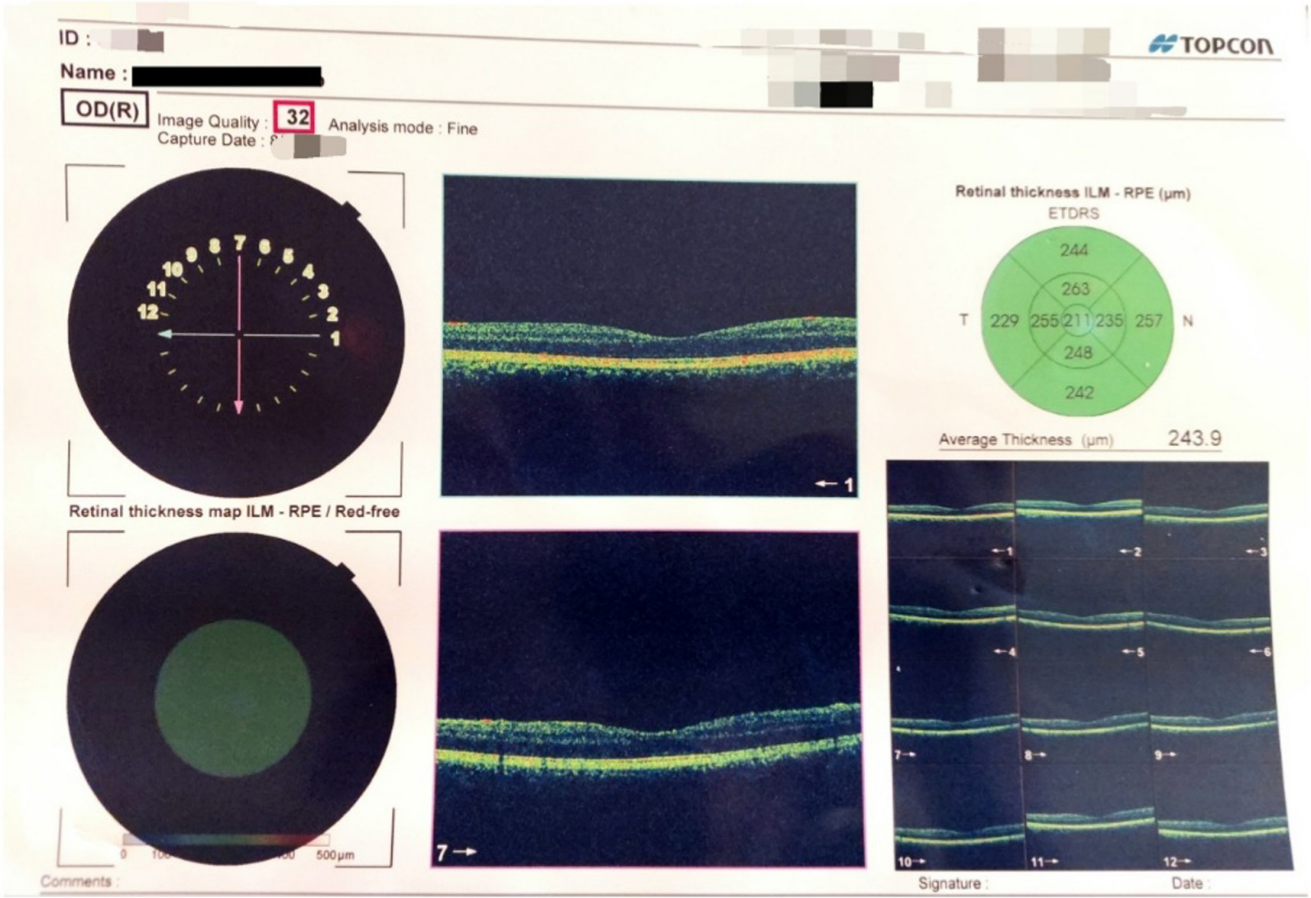
Ophthalmological examination revealed counting fingers in both eyes.

Ophthalmological examination revealed counting fingers in both eyes (Figure 1).

**FIGURE 2:**
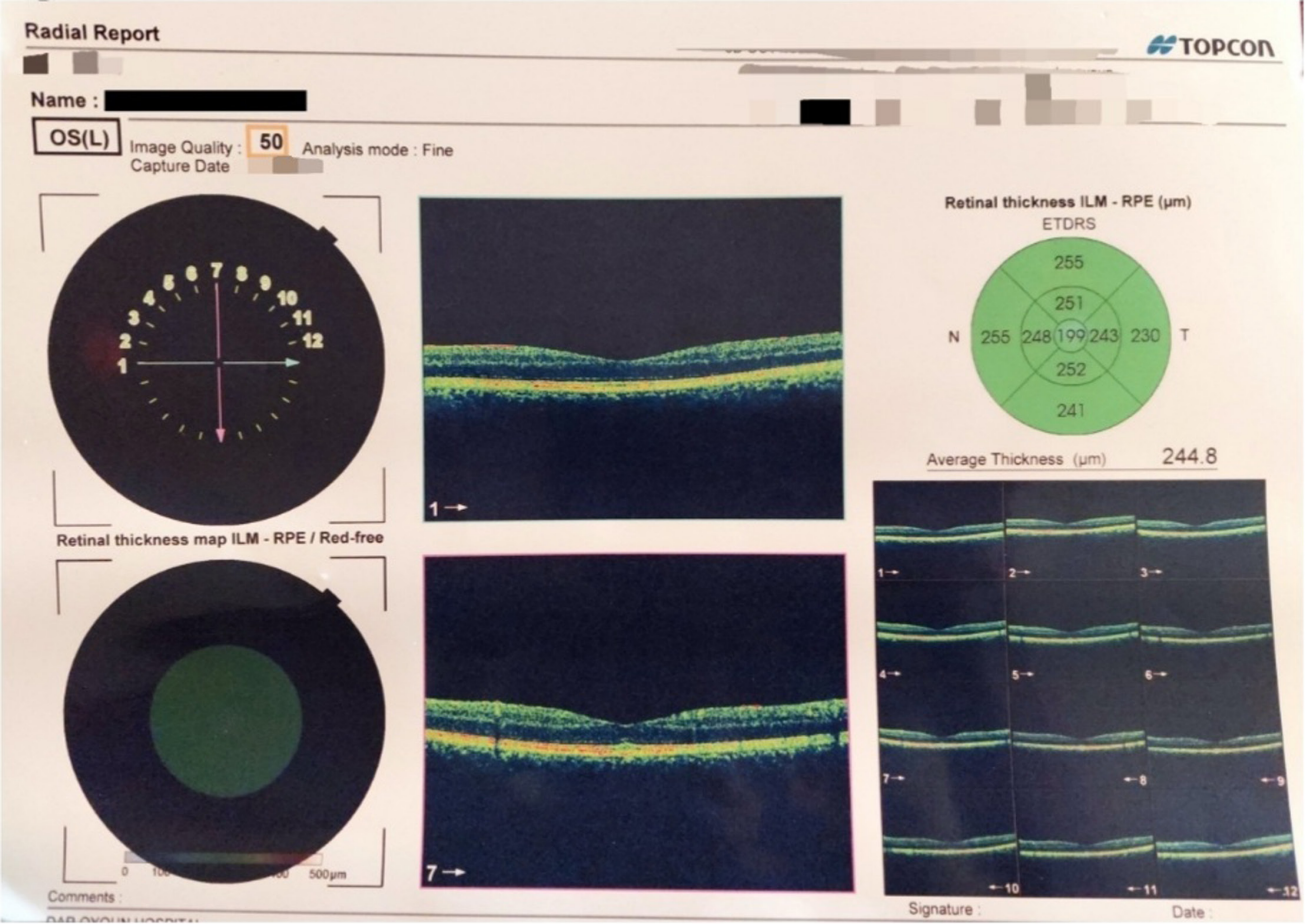
Visual field imaging (right eye).

**FIGURE 3:**
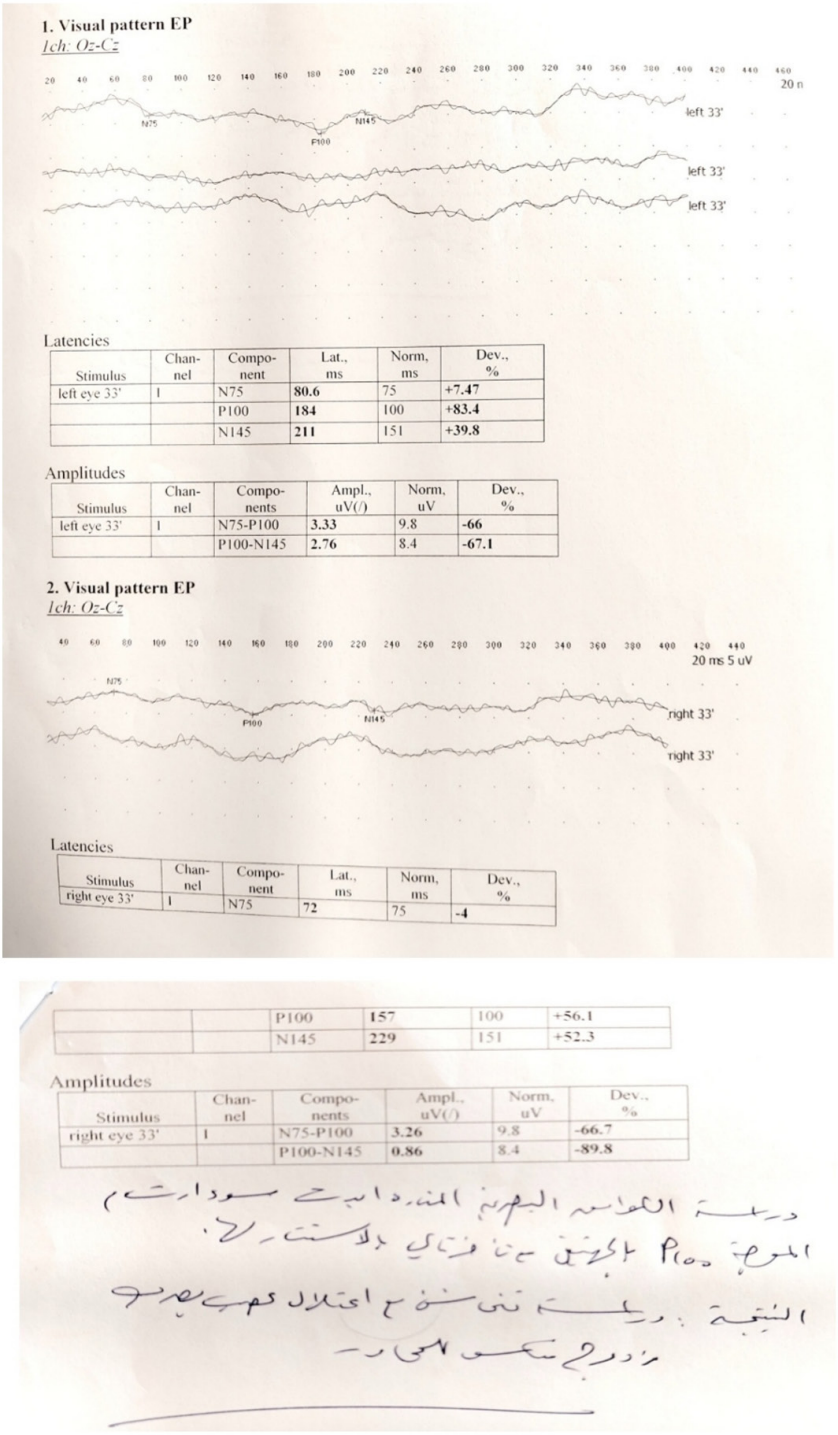
Visual field imaging (left eye).

The indirect light reflex was normal, but the afferent pupillary defect was positive. Bilateral optic disc edema was noted on fundoscopy. Visual field imaging is shown in Figures 2 and 3.

**FIGURE 4:**
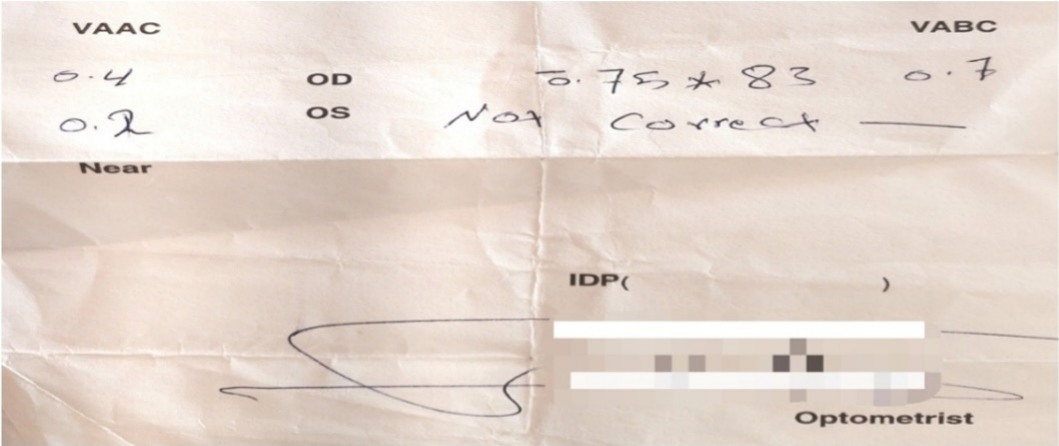
Visual evoked potentials (VEPs) revealed bilateral demyelinating optic neuritis.

Visual evoked potentials (VEPs) revealed bilateral demyelinating optic neuritis, as shown in Figure 4.

Laboratory tests including WBC, CRP, ESR, urea, creatinine, Vit B12, and liver enzymes were within normal ranges. The infectious and virology profiles including HIV, hepatitis C, hepatitis B, syphilis, TB, and leprosy were negative. The immune profile including ANA, anti‐phospholipid antibodies, C‐ANCA, P‐ANCA, and IgG4 was negative. Anti‐aquaporin 4 antibody and anti‐myelin oligodendrocyte glycoprotein antibody were also negatives. Cerebral spinal fluid analysis was normal.

**FIGURE 5:**
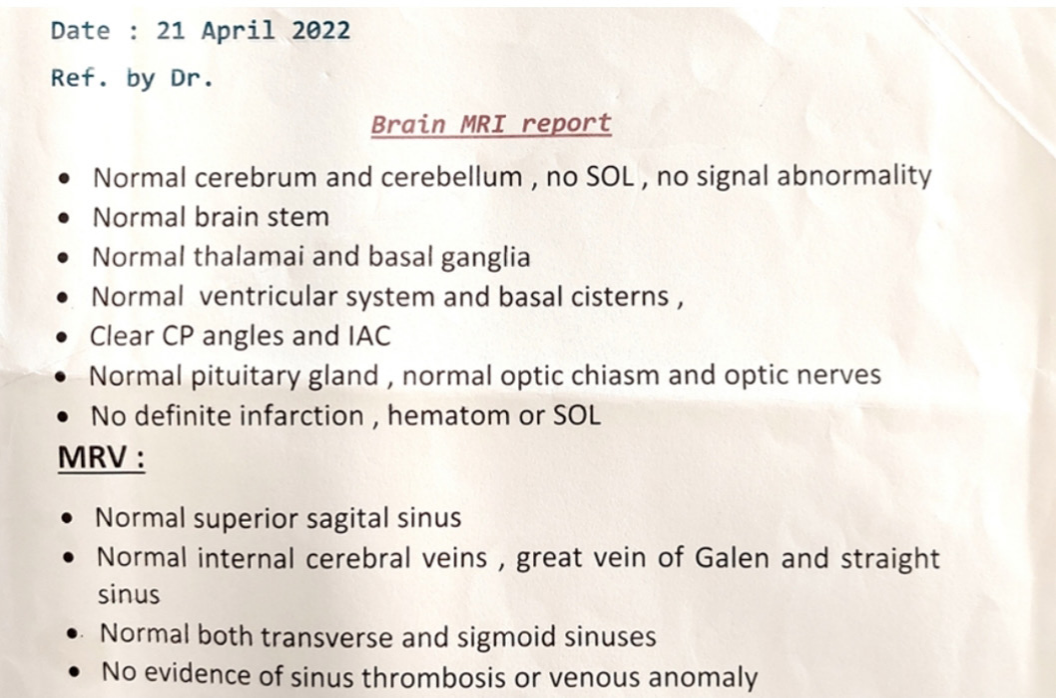
Brain MRI report.

Brain magnetic resonance imaging and magnetic resonance vein were normal, and no findings suggestive of demyelinating disease or compressive lesions were found (Figure 5).

Optic nerve MRI was normal.

Neck, thoracic, abdominal, and pelvic CT scans were normal.

He was treated with 1000 mg/IV/day methylprednisolone for 5 days followed by 70 mg/day oral prednisolone. Ophthalmological examination showed that disappearing of the bilateral optic disc swelling, without any improvement of visual capacity. The headache disappeared.

Another course of methylprednisolone pulse therapy for 5 days and 750 mg/IV/M2 cyclophosphamide every 15 days had been initiated, in addition to five courses of plasmapheresis. His visual function showed no improvement after the six doses of cyclophosphamide and 60 mg/day prednisone.

## 3 DISCUSSION

Many complications following vaccination including ocular manifestations had been seen. Uveitis, vision loss, papilledema, central serous retinopathy, central retinal vein occlusion, and others have been reported.^4^, ^5^ ON after vaccination against influenza, polio, hepatitis B, diphtheria, tetanus, and others giving rise to permanent visual loss in some cases has also been mentioned.^3^, ^6^

Molecular mimicry, superantigen stimulation, and bystander activation may play a role in the development of demyelinating lesions of the nervous systems.^7^ Immune complex may cause vascular damage, leading to perivascular inflammation, vascular permeability, and blood–brain barrier disruption, and the last event allows antibodies to enter the brain causing demyelination.^8^

The mechanism of ON following mRNA vaccination is still unclear. Increased serum cortisol, free extracellular mRNA, and polyethylene glycol may be the cause.^4^, ^9^

Here, an autoimmune response leading to bilateral ON was triggered by mRNA COVID‐19 vaccination, while it is difficult to prove the association with the causation of vaccines.^2^, ^3^, ^6^, ^7^

Critical visual loss occurs suddenly with pain, and only 0.4% of patients develop symptoms in both eyes simultaneously,^5^, ^7^, ^9^ as in our case. VEP studied the visual function, and magnetic resonance imaging considered as a sensitive indicator of demyelination in ON,^10^, ^11^ as in our patient.

A Cochrane review evaluating the beneficial effects of corticosteroids in terms of visual recovery, and visual field.

When these patients had poor response to steroids, immunotherapy, plasmapheresis, or intravenous immunoglobulin should be initiated at the earliest.^4^, ^5^, ^7^

The long‐term visual outcome of ON is good, although one in three patients remains visually impaired, and this is usually accompanied by more extensive lesions on magnetic resonance imaging and lower levels of VEP.^4^, ^5^, ^7^ In our case, the prognosis was very poor ended with blindness even there were no extensive lesions on magnetic resonance imaging.

## 4 CONCLUSION

Optic neuritis following COVID‐19 vaccination against rarely reported in the literature. Only a total of 8 reports on 9 patients have been published to date, 8 of 9 were females.^5^, ^7^, ^9^, ^11^ Our case is the third case of post‐COVID‐19 vaccination optic neuropathy in a male. The cases affect young females more, with improvement with IV methylprednisolone therapy for most of them, after^6‐^14 days after the COVID‐19 vaccination; meanwhile, our patient is a male who developed ON after 10 days of vaccination and did not respond to IV methylprednisolone therapy.

In conclusion, several cases of optic neuropathy have been reported, with good prognoses with treatments. Additional studies are recommended.

## AUTHOR CONTRIBUTIONS

**Raghad Tarcha:** Conceptualization; data curation; investigation; writing – original draft; writing – review and editing. **Afraa Ghazal:** Conceptualization; data curation; formal analysis; project administration; validation; writing – original draft. **Lama Al‐Darwish:** Investigation; supervision; validation; writing – original draft. **Hoda Abdoh:** Writing – original draft; writing – review and editing. **Maysoun Kudsi:** Project administration; supervision; writing – original draft; writing – review and editing.

## FUNDING INFORMATION

No funding.

## CONFLICT OF INTEREST STATEMENT

The authors have no conflicts of interest to declare.

## ETHICS STATEMENT

This case report was according to the Declaration of Helsinki. The ethical approval for this report was not necessary.

## CONSENT

Written informed consent was obtained from the patient to publish this report in accordance with the journal's patient consent policy.

## Data Availability

Not Applicable.
